# Deficiency of regulatory B cells in a house dust mite model of asthma

**DOI:** 10.1186/1479-5876-10-S3-P25

**Published:** 2012-11-28

**Authors:** Faouzi Braza, Julie Chesné, G Mahay, MA Cheminant, D Lair, K Botturi-Cavaillès, Antoine Magnan, Sophie Brouard

**Affiliations:** 1 Université de Nantes, France; 2 UMR_S1084, Institut de transplantation d’urologie et néphrologie, Nantes, France; 3 UMR_S 1087, Institut du Thorax, Nantes, France

## Introduction

Asthma is a chronic disorder leading to bronchial obstruction in response to inhaled allergen. It is associated with immune deregulation with specific expansion of Th_2_ and Th_17_ CD4^+^ T cells. Both T cell populations support B cells response by stimulating their proliferation, survival and IgE secretion. B cells are described for their effector functions but recently reports have described their regulatory role in autoimmune and inflammatory disorders. However, definitive identification has been challenging because regulatory B cells (Breg) are rare, do not have a specific marker, and express detectable IL-10 or TGF-β only upon *ex vivo* stimulation. In OVA asthma models, local inhalation tolerance [1], [2] and infections with helminthes [3], [4] induce the generation of regulatory B cells. But no physiological role of this population in the development of asthma has been described yet.

## Methods

Mice were sensitized on days 0, 7, 14 and 21 by percutaneous administration of HDM onto the ears. Intra-nasal challenges were performed on day 27 and 34 with 250 μg HDM. One day after each challenge, we realized by flow cytometry a complete B cell phenotyping in spleen and lungs.

Splenocytes and lung cells were isolated and stimulated *ex vivo* with LPS and PMA, ionomycin to induce IL-10 secretion by B cells.

## Results

No differential frequency was observed for all B cell populations in the spleen of HDM allergic mice, suggesting a normal B cell development. In contrast, HDM allergic mice exhibit a strong infiltration of CD19^+^ B cells in lungs and broncho-alveolar lavage after the second challenge. We found an increase of CD19 IgD^hi^ IgM^low^ B2 mature and CD19 IgD- IgM- switched memory B cells in the lung of HDM allergic compared to control mice. We looked at CD19^+^ IL-10^+^ CD1d^hi^ CD5^+^ CD21^+^ CD24^hi^ IgM^hi^ B cell population that has been shown to display regulatory properties in other situations. Whereas this population is present in spleen and lungs of HDM allergic mice, it produces less IL-10 than control after the first and the second challenge both in lung (vs control, p<0.01) and spleen (vs control, p<0.05).

## Conclusions

Our results strongly suggest a potential defect of regulatory B cells in the course of asthma. Future investigations will focus on their capacities to inhibit bronchial hyperreactivity and inflammatory responses.
